# Urticaria vasculitis in a child: a case report and literature review

**DOI:** 10.1002/ccr3.1027

**Published:** 2017-06-21

**Authors:** Adrián Imbernón‐Moya, Elena Vargas‐Laguna, Fernando Burgos, Eva Fernández‐Cogolludo, Antonio Aguilar‐Martínez, Miguel Ángel Gallego‐Valdés

**Affiliations:** ^1^ Department of Dermatology Hospital Universitario Severo Ochoa Avenida de Orellana 28911 Leganés Madrid Spain; ^2^ Department of Pathology Hospital Universitario Severo Ochoa Avenida de Orellana 28911 Leganés Madrid Spain

**Keywords:** Exanthem, inflammatory disorders, urticaria, vasculitis

## Abstract

Annular urticarial lesions in a child must establish a main differential diagnosis with urticaria multiforme, common urticaria, acute hemorrhagic edema of infancy, erythema marginatum, erythema annulare centrifugum, annular erythema in childhood, erythema multiforme, Sweet's syndrome, Schönlein‐Henoch purpura, erythematosus lupus, several systemic vasculitis, and serum sickness.

A 6‐year‐old child with no relevant medical history came to our clinic with itchy skin lesions located on her thighs. The lesions had appeared 2 weeks before in three‐day outbreaks appearing always at the same location. Spontaneous resolution without residual injury was achieved. No systemic symptoms or previous infectious diseases were associated. Her general condition was good. She denied the application of topical products or contact with local heat sources, neither sun exposure, previous sunburn, bites, nor contact with animals. No medication was taken before. Cutaneous examination revealed well‐defined, confluent, and erythematous papules and plaques, with annular arciform and polycyclic morphology with central clearing and purpuric edges. The lesions were located symmetrically on her anterior thighs giving an appearance of mirror watching lesions (Fig. 1).

**Figure 1:**
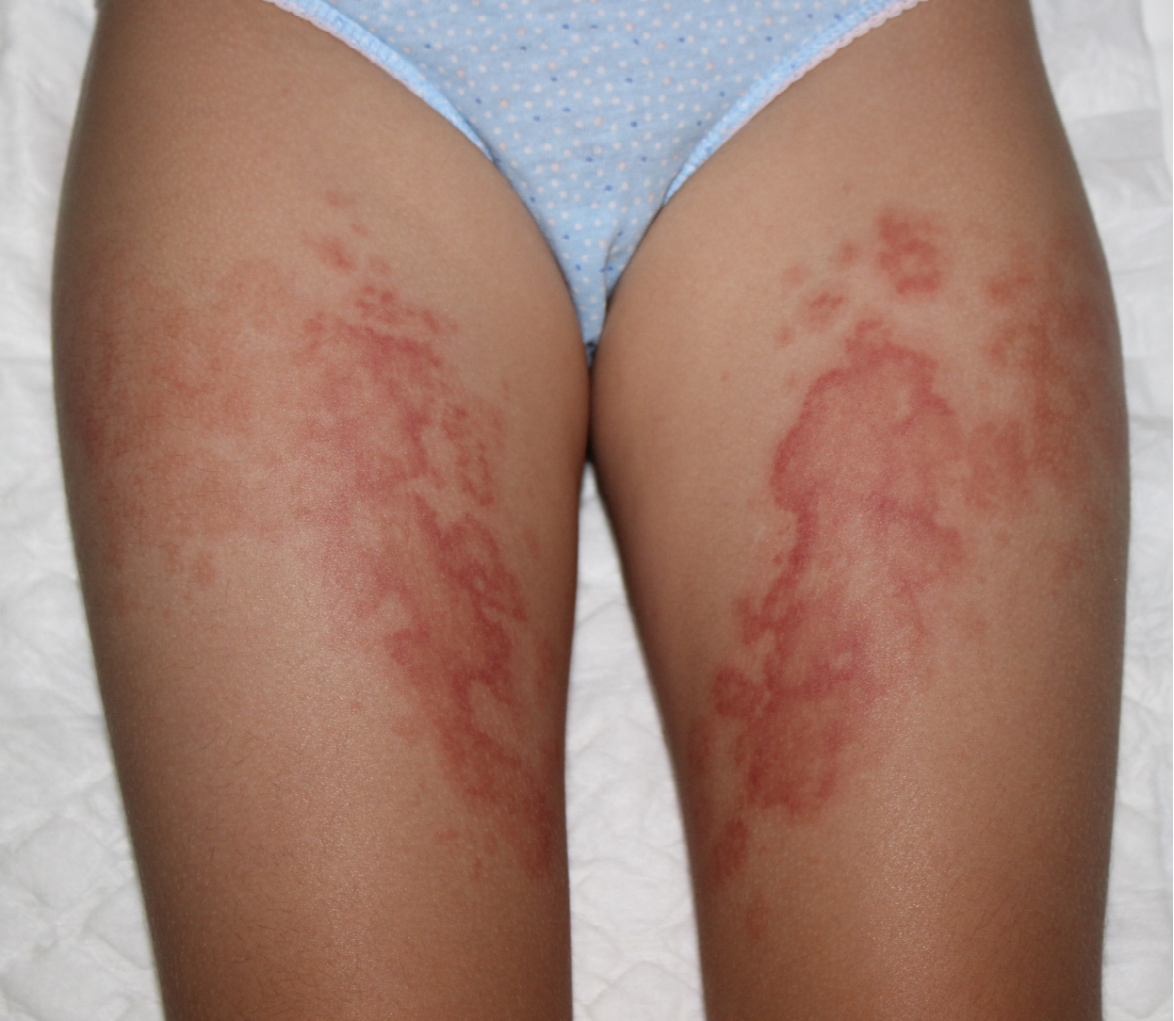
Polycyclic urticarial plaques with purpuric on her anterior thighs.

The following laboratory evaluations were within the normal ranges: biochemistry, urinalysis, complete blood cell count, hemostasis, erythrocyte sedimentation rate, C‐reactive protein, electrophoretic spectrum, immunoglobulin levels, complement (C3, C4, C1q), and rheumatoid factor. Subsequent antibody tests were negative: antinuclear, transglutaminase, anti‐double‐stranded DNA, antineutrophil cytoplasmic, anti‐Ro and anti‐La. Syphilis, HCV, HBV, HIV, CMV, EBV, and parvovirus B19 serology were negative. Chest radiographs were taken with no pathological findings. A skin biopsy was performed, and a leukocytoclastic vasculitis with edema in the papillary dermis was observed (Fig. 2). Direct immunofluorescence was negative.

**Figure 2:**
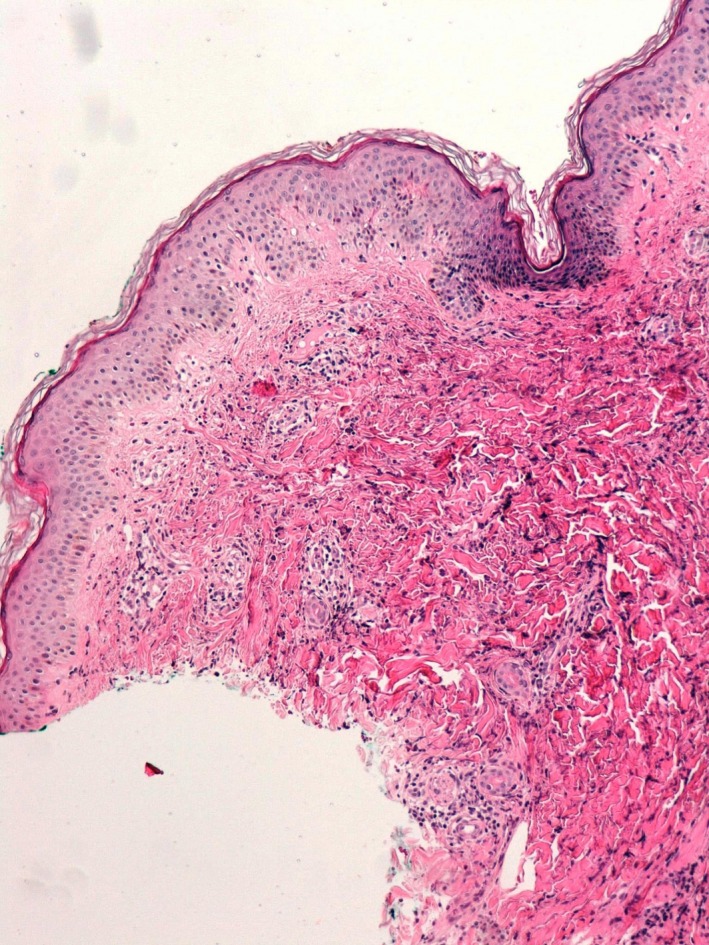
Edema in papillary dermal, perivascular lymphocytic infiltrates, leukocytoclasia, extravasation of erythrocytes, and interstitial eosinophils.

After 2 weeks, the disease was totally resolved without treatment. No scarring or residual hyperpigmentation remained. In the following 6 months, no new active skin lesions were detected.

Urticaria vasculitis (UV), first reported in 1973 by McDuffie 1, is a clinical–pathological variant of leukocytoclastic vasculitis that affects postcapillary venules. The prevalence is unknown and the incidence varies between 2% and 20%. Almost 60–80% of the cases occurs in women in their middle ages being rare in children 2, 3, 4, 5.

The mechanisms of action remain unknown; nevertheless, several authors support the hypothesis that the disease may be caused due to a three‐type hypersensitivity reaction against antigens not elucidated. These antigens may cause the development of circulating immune complexes, complement, and immunoreactants deposition on the walls of the blood vessels. The classical pathway of complement can also be activated by theses antigens 2, 4.

The skin lesions usually present as hives that can vary in size and typically persist for more than 24 h, with a mean duration of 3–4 days. They often spread transforming themselves in extensive plaques. Occasionally, residual hyperpigmentation remains. In most cases, UV is pruritic; however, pain, burning and tenderness may be associated. Skin lesions usually appear in anatomical areas of pressure and may have purpuric elements like petechiae and bruising. Angioedema occurs in up to 42% cases. Macular erythema, livedo reticularis, nodules, blisters, and Raynaud phenomenon rarely are observed 2, 3, 5.

The most common systemic symptoms are fever (10%), malaise, arthralgia, and/or transient and migratory arthritis (50%). A multisystem involvement could be present including involvement of the gastrointestinal tract (20%), nephritis (10%), chronic obstructive pulmonary disease and asthma (20%), hepatosplenomegaly, episcleritis, uveitis, and conjunctivitis. In the laboratory, evaluations of UV acute‐phase reactants are frequently increased. Hypocomplementemia, antinuclear, and anti‐C1q antibodies are associated with a poor prognosis of the disease and with a systemic involvement. Nevertheless, these findings are exceptional in childhood 2, 5, 6, 7.

Although UV is usually preceded by an upper respiratory tract infection in children, in adults the most common cause is idiopathic. However, some etiopathogenic factors have been reported as sun exposure, connective tissue disease, serum sickness, hematological disorders, solid tumors, infections (HBV, HCV, EBV, Borrelia burgdorferi), and abnormalities of complement and immunoglobulins. Several drugs have been described as possible precipitating factors, including cimetidine, nonsteroidal anti‐inflammatory drugs (NSAID), angiotensin‐converting enzyme inhibitors, fluoxetine, paroxetine, sodium valproate, diltiazem, procainamide, atenolol, thiazides, procarbazine, sulfonamides, ciprofloxacin, penicillins, and potassium iodide 2, 4, 5, 7, 8.

The diagnosis is confirmed by the histopathological findings that are characterized by swelling and necrosis of endothelial cells, a perivascular inflammatory infiltrate mainly neutrophilic, extravasation of red blood cells, perivascular leukocytoclasia, and interstitial fibrinoid deposit. Direct immunofluorescence can be found in 70–80% of cases with a linear or granular deposit of immunoglobulins, complement (C3), and/or fibrinogen in the vascular endothelium and/or in the basement membrane. The presence of deposit in the basement membrane associated with hypocomplementemia recommends to rule out systemic erythematosus lupus 2, 4, 7, 8.

Annular urticarial lesions in a child must establish a differential diagnosis with urticaria multiforme, common urticaria, acute hemorrhagic edema of infancy, erythema marginatum, erythema annulare centrifugum, annular erythema in childhood, erythema multiforme, Sweet's syndrome, Schönlein‐Henoch purpura, erythematosus lupus, several systemic vasculitis, and serum sickness 5, 6, 9, 10.

Treatment options include antihistamines and NSAID in mild cases. In severe cases, colchicine, dapsone, antimalarials, systemic corticosteroids, azathioprine, cyclosporine, methotrexate, mycophenolate mofetil, intravenous immunoglobulin, plasmapheresis, and rituximab have been used with varying effectiveness. However, the course of the disease is usually benign with a history of lesions developing in 3–5 days outbreaks in a 3‐year period 2, 4, 5, 6, 7.

## Authorship

AI‐M: drafted the article and approved the final version. EV‐L, FB, EF‐C, AA‐M, and MÁG‐V: revised and approved the final version.

## Conflict of Interest

The authors have no conflict of interest to declare.
